# Periodicals in Exchange

**Published:** 1850-10-01

**Authors:** 


### PERIODICALS IN EXCHANGE.

The British and Foreign Medical and Chirurgical Review.
Provincial Medical and Surgical Journal.
Dublin Medical Press.
The Massachusetts Monthly Review.
The Ecclesiologist.
London Journal of New Patent Inventions.
New York Journal of Medicine.
Annales Medicales Psychologiques.
Monthly Journal of Medical Science.
The American Journal of Medical Science, edited by Dr. Hays.
The Quarterly Journal of Education.
Dr. Ranking's Half-Yearly Abstract of the Medical Sciences. [A very able publication, containing the cream of medical literature, and selected and arranged with great judgment.]

